# Shy Like Me: Exploring Links Between Coaches’ Shyness and Beliefs About Shy and Exuberant Children in a Team Sports Context

**DOI:** 10.1177/01937235251401868

**Published:** 2025-12-02

**Authors:** Megan DeGroot, Kristen A. Archbell, Robert J. Coplan

**Affiliations:** 1 Department of Psychology, 414990 Carleton University, Ottawa, Canada; 2 School of Pharmacy, 8430 University of Waterloo, Waterloo, Canada

**Keywords:** Shyness, coaches, sports, beliefs, youth

## Abstract

This study examined links between coaches’ shyness and their perceptions of shy and verbally exuberant children in a team sports context. Participants were a community sample of *N* = 465 adults (*n* *=* 379 males; *n* *=* 86 females) aged 18–78 years (*M* = 43.48 years, *SD* = 9.96) with previous experience coaching children in team sports. Measures included demographic questions, respondents’ self-reported shyness, responses to vignettes describing hypothetical shy and verbally exuberant children in a team sports context, and an assessment of potential problems for shy athletes. Among the results, coaches’ shyness was significantly associated across both vignettes with overall worry (*F*(1, 406) = 5.40, *p* = .021), greater anticipation that these children would experience peer difficulties (*F*(1, 401) = 4.39, *p* = .037), and lower efficacy for working with these children (*F*(1, 405) = 6.12, *p* = .014). These findings suggest that shyness among coaches may be manifested as higher threat perception and lower confidence in the context of children's behavioral management. As a result, children with different behavioral tendencies may not reap all the benefits that team sports participation can provide. This highlights the importance of sports coaches’ personality traits on the outcomes for shy and verbally exuberant team members and the need for support for shy coaches.

Team sports provide numerous benefits to children and adolescents (Eime et al., 2013), including promoting the development of physical abilities (Morano et al., 2011) and reducing internalizing problems (Aumètre & Poulin, 2018; Graupensperger et al., 2021; White-Gosselin et al., 2023). There is also some evidence to suggest that participation in sports teams can be particularly beneficial to the socio-emotional development of *shy* children, including increasing self-esteem and decreasing social anxiety symptoms (Findlay & Coplan, 2008). Despite these positive benefits, shy children are less likely than their non-shy peers to participate in organized sports activities (Aumètre & Poulin, 2016). Moreover, when they do participate, shy children are most likely to engage in individual sports (e.g., swimming, gymnastics), which feature significantly more independence than team sports (e.g., hockey, soccer; Miller, 2012). Because individual sports do not offer the experience of working in tandem with other peers, they may not offer children the same social benefits that team sports provide (Eime et al., 2013).

Sports coaches have a unique opportunity to support the socio-emotional development of the children on their teams through the facilitation of a positive team environment (Bailey et al., 2013; Claringbould et al., 2014). In this regard, researchers have explored how individual differences in coaches’ characteristics are associated with their beliefs and behaviors, including personality traits (Jackson et al., 2011), coaching experience (Feltz et al., 1999), and coaching philosophy (Camiré et al., 2014). However, to date, we are not aware of any previous studies that have specifically explored the implications of coaches’ shyness in this regard. There is at least some previous research to suggest that shyness among teachers is related to their attitudes and behaviors towards shy students. For example, as compared to their more sociable counterparts, shy teachers tend to be more understanding towards shy students and see their lack of participation in class as a reflection of a lack of confidence rather than a lack of ability (Coplan et al., 2011). We speculated that coaches’ shyness might also be associated with their beliefs about shy versus verbally exuberant (i.e., sociable/talkative) children on their teams. Accordingly, the primary purpose of the current study was to explore the relations between coaches’ self-reported shyness and their beliefs and reactions to hypothetical shy and verbally exuberant child and adolescents in a team sports context.

## Overview of Shyness

Shyness is a temperamental trait characterized by heightened experiences of wariness and self-consciousness in novel or social situations (Rubin et al., 2009). In early childhood, shyness emerges as a wariness and fear of social interactions, particularly in novel contexts (Buss, 1986). Among older children, shyness also comes to encompass embarrassment in situations of perceived evaluation by others (Eggum-Wilkens et al., 2015). Shyness is quite stable across childhood, adolescence, and into adulthood (Karevold et al., 2012; Tang et al., 2017). In social contexts such as school, shy students tend to be less engaged in the classroom environment (Hughes & Coplan, 2010) and are prone to withdrawing from opportunities for peer engagement (Kalutskaya et al., 2015). This lack of communicative engagement may lead teachers to believe that these students are less academically adept (Hughes & Coplan, 2010). Teachers also tend to worry more about shyness and anticipate negative consequences for their shy students as compared to other types of social behaviors and misbehaviors in the classroom (Coplan et al., 2011; Deng et al., 2021).

When shyness persists into adulthood, it is associated with loneliness, lower self-esteem, and poorer family functioning (Schmidt et al., 2017). As compared to their more sociable counterparts, shy adults also report lower self-efficacy and self-confidence (Bober et al., 2022), as well as heightened threat perception and anticipation of negative outcomes (Tang et al., 2017). It has been argued that these characteristics of shy adults impair social functioning and can act as risk factors for more serious anxiety problems (Bar-Haim et al., 2007).

### Shyness in the Context of Sports

Sports participation is beneficial for youth across various developmental domains, such as social and physical development (Eime et al., 2013). However, it has been suggested that sports participation, particularly *team* sports, may be especially important for shy children because it provides exposure to new social contexts, promotes the development of social skills, and potentially helps reduce anxiety (Findlay & Coplan, 2008). Notwithstanding, only a handful of previous studies have specifically explored the implications of shyness in a sports context.

To begin, there is some evidence to suggest that shy children are less likely than their more sociable counterparts to engage in extracurricular sporting activities (Aumètre & Poulin, 2016; Miller, 2012). Youth involved in sports tend to report greater negative peer experiences compared to youth involved in other activities, including pressure from peers to engage in negative behaviors such as illicit substance use and the promotion of negative social norms (Hansen et al., 2003). Such experiences may deter shy youth from participating out of worry about negative peer perceptions or evaluations. The stresses of competition may also evoke anxiety among shy athletes (Kingsbury et al., 2011; Prakash & Coplan, 2003). However, in general, teachers tend to rate children who participate in team sports as more socially adept, and less shy and socially withdrawn than children who do not participate in team sports (McHale et al., 2005). This speaks to the lack of understanding that shy children and their families may have regarding the benefits that team sports participation can have for socio-emotional development.

Notwithstanding, when shy children do participate in sports, they do appear to reap some benefits. For example, Findlay and Coplan (2008) found that shy elementary school children who participated in team sports reported a significant decrease in social anxiety symptoms 1 year later. Team sport participation has also been found to decrease feelings of loneliness through the increase of perceived social competence, even when controlling for shyness (Haugen et al., 2013). These findings suggest that team sports provide shy children with opportunities to expand their social networks and develop their social skills, which may help decrease some of the negative outcomes associated with shyness.

### Coaches and Shy Children

Our review of the literature uncovered only two empirical studies where researchers have specifically examined coaches’ beliefs about shy youth on their teams. Kirkpatrick et al. (2020) explored the attitudes and beliefs towards shy youth athletes of 447 undergraduate students with coaching experience. Participants were asked to respond to vignettes depicting children displaying different social/non-social behaviors in a team sports context. The vignettes included depictions of a shy child and a *verbally exuberant* child (characterized by high social approach, low social anxiety, and high dominance or disruption in social situations; Fox et al., 2001). After reading each vignette, participants were asked to indicate how they would react emotionally, their perceived implications of each child's behavior (for their development and for their performance on the team), and their anticipated peer responses to each child. They found that coaches reported the most worry about the hypothetical shy athlete, the most anger towards the verbally exuberant athlete, and anticipated that shy children would experience the greatest peer difficulties and negative developmental outcomes.

Nguyen et al. (2021) explored coaches’ beliefs about the benefits for and the contributions made by shy youth athletes on sports teams in a community sample of 496 adults with coaching experience. Participants responded to open-ended questions asking about the potential benefits that shy children may experience from team sports participation, as well as what unique contributions shy players may make to a sports team. Results indicated that coaches tended to believe that sports participation is beneficial for shy children in terms of fostering their social skills, increasing their sense of self-esteem and belongingness, and helping to overcome shyness. Coaches also reported that shy children make equal contributions to the team as non-shy children but can also uniquely contribute to the team as “quiet leaders.” Taken together, the initial findings of these two studies suggest that although coaches do not hold particularly negative views of shy team members, they are aware of the potential developmental consequences that shyness may hold for their athletes.

## The Current Study

There has only been limited research exploring how shy children are perceived by coaches in a team sports context (Kirkpatrick et al., 2020; Nguyen et al., 2021). Moreover, to date, no research has considered the influence of the coaches’ *own* shyness on their perceptions of shy youth athletes. Given that coaches play a prominent role in youth athletes’ enjoyment, effort, and feelings of competence in their sport (Chan et al., 2012), it is important to consider a variety of potential factors that affect coaches’ perceptions of their athletes.

The primary goal of the current study was to explore links between coaches’ shyness and their beliefs about hypothetical shy and exuberant youth athletes. Drawing upon previous research among coaches and teachers, it was hypothesized that overall, coaches would worry more about, anticipate negative social outcomes, and feel less prepared for hypothetical shy youth athletes compared to verbally exuberant athletes. It may be that specific characteristics of the classroom (e.g., having to sit quietly for extended periods of time) make it a more challenging context for exuberant students (Coplan et al., 2011) than the sports arena.

In terms of the associations between coaches’ *own* shyness and their beliefs towards shy and exuberant athletes, predictions were more tentative in nature. To our knowledge, there have been no previous studies of shyness among coaches. However, we uncovered two previous studies examining this trait among *teachers*. Coplan et al. (2011) presented 275 elementary school *teachers* with hypothetical vignettes depicting “shy,” “verbally exuberant,” and “average” students. Of particular interest, both shy and non-shy teachers believed that hypothetical shy students would perform less well academically as compared to their non-shy classmates. However, non-shy teachers also viewed shy children as less intelligent than their non-shy peers, whereas shy teachers did not. The authors suggest that teachers who are higher in shyness themselves may display a greater ability to empathize with the experiences of shy students and understand that their academic under-performance may be due to other factors besides intelligence.

Similarly, Deng et al. (2021) asked a sample of 335 university students training to become elementary school teachers to respond to hypothetical vignettes depicting a “shy,” “verbally exuberant,” and “average” child in the classroom. Results indicated that shy pre-service teachers reported lower self-efficacy for working with both shy and verbally exuberant children compared to their non-shy counterparts. This finding is consistent with previous research linking shyness in adults to less self-efficacy, confidence, and self-esteem (Bober et al., 2022). Drawing upon this research, it was tentatively postulated that coaches’ shyness would be associated with greater worry, anticipation of negative social outcomes, and lower feelings of preparedness for hypothetical shy youth athletes.

Finally, we also explored gender and age effects pertaining to the hypothetical child. It has been argued that shyness is viewed as more acceptable among girls compared to boys because it violates gender stereotypes more for boys, who are depicted as more dominant and assertive, than girls, who are characterized as more sensitive and submissive (Doey et al., 2014). In this regard, coaches might be expected to have more negative attitudes toward boy versus girl shy youth athletes. Shyness may be considered more developmentally acceptable in early childhood compared to later childhood (MacGowan & Schmidt, 2024). Older children may be expected to have developed the socio-emotional skills necessary to engage with others without shyness influencing their behavior. In this way, we speculated that coaches might display more negative attitudes toward older versus younger shy youth athletes.

## Method

### Participants

Participants were *N* = 465 adults (*n* *=* 379 males; *n* *=* 86 females) aged 18 to 78 years (*M_age_* = 43.48 years, *SD* = 9.96) who had coached a youth sports team in the last 12 months. The average amount of coaching experience was 13.17 years (*SD* = 10.32). The age groups the participants coached included 4.7% 6 years or younger, 8.0% 7 to 8 years old, 11.6% 9 to 10 years old, 19.8% 11 to 12 years old, 33.8% 13 to 15 years old, and 21.9% 16 to 19 years old. Participants reported coaching boys (*n* = 183, 39.4%), girls (*n* = 112, 24.1%), or both (*n* = 170, 36.6%). The sample was predominately Caucasian (*n* = 441, 94.8%). Coaches’ education levels included 12.9% high school, 21.5% college, 45.6% university, 16.1% graduate, and 3.9% other education levels. Participants coached teams in a variety of locations across Canada, the most frequent being in Ontario, Alberta, and British Columbia.

Participants reported coaching a variety of sport types, including hockey (36.6%), soccer (18.5%), football (13.1%), basketball (6.0%), baseball (5.8%), ringette (5.6%), lacrosse (2.8%), rugby (9.0%), and others (10.8%). The sports levels the participants coached included 62.8% competitive, 35.5% recreational, and 1.7% other levels.

### Procedure

Ethics approval for the current study was provided ***blinded for peer review***. Participants were recruited across Canada using both a snowball sampling method and participation invitations to sports organizations. Data from the current study were obtained from a larger study investigating coaches’ perceptions and responses towards hypothetical children in a sports team context (***blinded for peer review***). Participants were presented with two hypothetical vignettes depicting children displaying various social behaviors in a team sports context. Participants then responded to a series of questionnaires on their likely responses towards each hypothetical child.

### Measures

#### Coaches’ Shyness

Participants’ shyness was assessed using the *Revised Cheek and Buss Shyness Scale Short Form* (RCBS, Cheek, 1983). This 13-item scale asks participants to rank items based on how characteristic they believe that item to be to their own feelings and behaviors on a 5-point scale (1 = “very uncharacteristic or untrue,” 5 = “very characteristic or true”). Items include “I feel tense when I’m with people I don’t know well,” “It is hard for me to act natural when I am meeting new people,” and “I feel inhibited in social situations.” The RCBS is one of the most commonly used measures of adult shyness, has excellent psychometric properties, including a consistent factor structure, as well as evidence of reliability and validity (for reviews, see: Crozier, 2005; Hopko et al., 2005). For example, in terms of construct validity, RCBS shyness scores have been shown to be significantly associated with lower self-esteem (peer difficulties and indices of internalizing problems (e.g., lower self-esteem, worry, and anxiety) (Bober et al., 2022; MacGowan & Schmidt, 2024). The shyness scale was found to have good internal reliability in the current sample (α = .86).

#### Hypothetical Vignettes

Participants were presented with two hypothetical scenarios, one depicting a child engaging in shy behavior and one depicting a child engaging in verbally exuberant behavior in a team sports context. Participants who indicated that they coached boys were assigned to the boy vignette conditions, while participants who indicated they coached girls or mixed-gender teams were assigned to the girl vignette conditions. The shy and verbally exuberant scenarios were presented to participants in a random order. After reading each vignette, participants were asked to imagine that this was a child on their team and answered a series of questions regarding how they would respond to the hypothetical child. We used the wording for the vignettes and follow-up questions developed and validate by Kirkpatrick et al. (2020), who adapted the measure for the sports context from similar assessments previously used with teachers (e.g., Coplan et al., 2011) and parents (e.g., Coplan et al., 2002; see Appendix A for the text of both vignettes). Kirkpatrick et al. (2020) previously demonstrated good psychometric properties for this measure and evidence of validity. For example, in their sample of undergraduate students with coaching experience, participants reported that as compared to hypothetical exuberant children, in response to hypothetical shy children they would: (1) be less likely to intervene to stop the behavior; (2) respond using less controlling, more indirect, and more autonomous supportive strategies; (3) feel less angry but more worried; and (4) anticipate more negative peer responses, negative implications for development, and negative implications for team performance.

The first set of items assessed the participants’ *emotional responses* towards each child's behavior on a 5-point scale (1 = “not at all,” 5 = “very strongly”). Participants were asked to respond to single items regarding the likelihood that they would be worried and the likelihood that they would be angry if a child on their own team was exhibiting behavior similar to the child in each hypothetical vignette.

The second set of items assessed the participants’ beliefs regarding anticipated *peer problems* for each hypothetical child on a sports team on a 5-point scale (1 = “very unlikely,” 5 = “very likely”). Participants were asked to respond to their belief in the likelihood that the child in each vignette will be well-liked by teammates (reverse-scored), excluded by teammates, ignored by teammates, and a leader on the team (reverse-scored). Results from principal components factor analysis indicated one-factor solutions for both the shyness vignette (Eigenvalue = 2.151 accounting for 71.72 percent of the variance, factor loadings ranging from .71–91) and the exuberant vignette (Eigenvalue = 2.131 accounting for 71.05 percent of the variance, factor loadings ranging from .76–91). Items were therefore aggregated to represent anticipated peer problems for the shyness (α = 0.68) and verbal exuberance vignettes (α = 0.75).

Finally, a single item assessed the participants’ *self-efficacy.* Participants were asked to rate on a 5-point scale (1 = “not at all,” 5 = “very well”) how well prepared they believed they would be to work with a child who was exhibiting behavior similar to the child in each hypothetical vignette.

#### Potential Problems for Shy Athletes

Participants also completed the newly developed *Potential Problems for Shy Athletes* (PPSA) scale, created for this study to assess coaches’ beliefs about the challenges faced by shy children in the context of team sports. Participants were prompted with: “How often do shy athletes on your team have problems with the following?”. The original pool of 8-item was created based on previous theory and research on shy children in the context of sports (Findlay & Coplan, 2008; Kirkpatrick et al., 2020; Miller, 2012; Nguyen et al., 2021). Item content related to peer problems, emotional problems, and problems more specific to the team sports context. Items were rated on a 5-point Likert scale (from 1 = “never” to 5 = “always”).

Results from Principal Axis Factoring with Promax rotation indicated a two-factor solution, accounting for 46.41 percent of the variance (all factor loadings > .40). The first factor was labeled *socio-emotional problems* (5 items, α = .76) because it included items pertaining to peer difficulties (e.g., “getting teased by other team members”) and symptoms of internalizing problems (“having low self-esteem”). The second factor was labeled *sports problems* (3 items, α = .73) and because it was comprised of related to issues with athletic skills (e.g., “performing up to skill level”) and being part of a team (e.g., “working as part of a team”). The complete measure is displayed in Appendix B.

### Overview of Analyses

Data were first cleaned and assumptions tested. Preliminary analyses were then conducted, including calculation of descriptive statistics and correlations among study variables. The primary analyses included a series of ANCOVAs to compute the predicted effects of vignette (Shy vs. Exuberant), gender of child (Boy vs. Girl), coaches’ shyness (tested by including it as a covariate), and child age (tested by including it as a covariate) for each of the vignette response variables and potential problems for shy athletes.

## Results

### Preliminary Analyses

Descriptive statistics and intercorrelations among all the study variables are presented in Table 1. Overall, there were very low rates of missing data across the study variables, with only two variables evidencing > 5% missing data (shyness = 11.12%; anger response to the shy vignette = 5.33%). Results from Little's MCAR test indicated that data was missing “completely at random.” As well, normality assumptions for some variables were violated as per the Shapiro–Wilk test. However, with a sample size > 100 for each variable, ANCOVA is robust enough to handle such violations as per the Central Limit Theorem (Ghasemi & Zahediasl, 2012). Examination of boxplots revealed a few outliers for each of our study variables, further examination of Cook's Distance and Leverage values indicated that no outliers were having undue influence in the model. As such, outliers were not removed.

**Table 1.** Descriptive Statistics and Zero-Order Correlations for all Study Variables.

Variable	*N*	*M*	*SD*	1	2	3	4	5	6	7	8	9	10
1. Coach shyness^a^	413	27.37	8.29	—	−.01	−.01	.09	−.11*	.12*	.09	.10	−.13**	.04
2. Age group^b^	464	4.36	1.40		—	.07	.07	.06	.03	.02	.04	.08	.04
*Shy vignette*													
3. Anger^a^	440	1.12	0.40			—	.18**	−.05	−.01	.11	.08	.05	−.02
4. Worry^a^	444	2.09	0.84				—	−.01	.12*	.25**	.27**	−.03	.14**
5. Preparedness^a^	444	3.96	0.86					-	−.25**	−.15**	.00	.60**	−.16**
6. Perceived peer problems^c^	443	0.001	0.84						—	.11*	−.04	−.13**	.35**
*Exuberant vignette*													
7. Anger^a^	450	1.65	0.86							—	.28*	−.14**	.23**
8. Worry^a^	451	1.58	0.79								—	−.12**	.19**
9. Preparedness^a^	452	4.13	0.75									—	−.16**
10. Perceived peer problems^c^	448	0.001	0.84										—

*p < .05; **p < .01.

a Rated on a 1–5 scale.

b 1 = <6 years old, 2 = 7–8 years old, 3 = 9–10 years old, 4 = 11–12 years old, 5 = 13–15 years old, 6 = 16–19 years old.

c *z-*score.

Preliminary analyses revealed a significant and negative correlation between coaches’ shyness and years of coaching experience (*r* = −.095, *p* = .047). Accordingly, this variable was controlled for in subsequent analyses. There was no significant difference in shyness among those who coached recreational (*M* = 28.06, *SD* = 7.44) versus competitive teams (*M* = 29.91, *SD* = 8.65; *t*(404) = 1.36, *p* = .175)^1^.

### Responses to Hypothetical Vignettes

The goal of the next set of analyses was to compare coaches’ responses to the vignettes depicting shy versus exuberant youth athletes and examine the effects of coaches’ shyness. To accomplish this goal, a series of ANCOVAs was computed, with Vignette (Shy vs. Exuberant) serving as a within-subjects variable, and Gender of Child (Boy vs. Girl) as a between-subjects variable. To test their predicted effects, coaches’ shyness and age were included as covariates. To control for its association with shyness, Coaching Experience was also included as a covariate. Separate ANCOVAs were computed for each of the dependent variables. Results are presented in Table 2.

**Table 2.** ANCOVAs Testing Effects of Child Vignette, Coaches Shyness, Age Group, and Child Gender.

Dependent variable
Worry	*F*	*p*	*ηp2*
Vignette	6.10	.014	.015
Shyness	5.40	.021	.013
Age group	0.67	.413	.002
Child gender	1.08	.299	.003
Coaching experience	1.83	.177	.004
Vignette** × **shyness	0.24	.627	.001
Vignette** × **age group	1.29	.257	.003
Vignette** × **child gender	6.62	.010	.016
Vignette** × **coaching experience	4.88	.028	.012

Anger	*F*	*p*	*ηp2*
Vignette	2.87	.091	.007
Shyness	1.75	.187	.004
Age group	1.45	.230	.004
Child gender	0.00	.994	.000
Coaching experience	0.82	.366	.002
Vignette** × **shyness	2.82	.094	.007
Vignette** × **age group	0.00	.958	.000
Vignette** × **child gender	1.29	.257	.003
Vignette** × **coaching experience	2.24	.135	.006

Peer problems	*F*	*p*	*ηp2*
Vignette	1.38	.241	.003
Shyness	4.39	.037	.011
Age group	1.02	.313	.003
Child gender	2.43	.120	.006
Vignette** × **shyness	2.24	.136	.006
Vignette** × **age group	0.14	.708	.000
Vignette** × **child gender	0.66	.417	.002
Vignette** × **coaching experience	0.13	.714	.000

Self-efficacy	*F*	*p*	*ηp2*
Vignette	0.60	.441	.001
Shyness	6.12	.014	.015
Age group	0.18	.672	.000
Child gender	1.45	.230	.004
Coaching experience	8.93	.003	.022
Vignette** × **shyness	0.04	.847	.000
Vignette** × **age group	0.28	.600	.001
Vignette** × **child gender	0.30	.582	.001
Vignette** × **coaching experience	0.35	.556	.001

For *worry,* results indicated significant main effects of coaches’ shyness and vignette. Overall, coaches were more likely to worry about hypothetical shy children (*M* *=* 2.08, SD = 0.84) than hypothetical verbally exuberant children (*M* *=* 1.57, *SD* = 0.76; *F*(1, 406) = 6.10, *p* = .014, *ηp2* = .015). Coaches’ shyness was significantly and positively associated with feelings of worry across both vignettes while controlling for child gender, child age, and coaching experience (*F*(1, 406) = 5.40, *p* = .021, *ηp2* = .010). The main effects of child gender, child age, and coaching experience were not significant. In terms of interaction effects, a significant vignette × child gender interaction was found. Follow-up simple effects analyses revealed that after controlling for child age and coaching experience, coaches worried more about hypothetical shy girls (*M* = 2.19, *SD* = 0.86) than hypothetical shy boys (*M* = 1.99, *SD* = 0.81; *F*(1, 406) = 6.62, *p* = .010, *ηp2* = .016).

For *anger,* results indicated no significant main effects or interaction effects for any of the independent variables.

For *beliefs of peer problems,* results indicated a significant main effect of coach shyness. Coaches’ shyness was significantly and positively associated with the belief that both hypothetical shy and verbally exuberant children will experience peer problems, while controlling for *c*hild gender, child age, and coaching experience (*F*(1, 401) = 4.39, *p* = .037, *ηp2* = .011). The main effects of child gender, child age, and coaching experience were not significant, and no significant interaction effects were found.

For *self-efficacy* results indicated a main effect of coaches’ shyness and coaching experience. Coaches’ shyness was significantly and negatively associated with feeling prepared for working with both hypothetical shy and verbally exuberant children, while controlling for child gender, child age, and coaching experience (*F*(1, 405) = 6.12, *p* = .014, *ηp2* = .015). In addition, coaching experience was significantly and positively associated with self-efficacy across both vignettes (*F*(1, 405) = 8.93, *p* = .003, *ηp2* = .022). The main effects of child age and child gender were not significant and no significant interaction effects were found.

### Potential Problems for Shy Athletes

The final set of analyses explored the links between coaches’ shyness and their anticipation of potential problems specific to shy athletes. Two hierarchical linear regressions were conducted to predict coaches’ beliefs about shy athletes facing *socio-emotional* problems and *sports-related* problems. For both equations, coaching experience was entered at Step 1 as a control variable, the “main effect” variables of coaches’ shyness, child gender, and child age were entered at Step 2, and the conceptually relevant interaction terms (i.e., coaches’ shyness** × **child age; coaches’ shyness** × **child gender) were entered at Step 3.

Results for *beliefs of socio-emotional problems* are presented in Table 3. The linear regression indicated a positive association between coaches’ shyness and their belief that shy athletes will experience socio-emotional problems in the team sports context, while holding child gender and age coached constant (*b* *=* .013, *p* < .001). That is, coaches’ shyness predicted the belief that shy athletes will experience socio-emotional problems. No other significant main or interactions effects were found.

**Table 3.** Hierarchical Regression Results for Beliefs of Socio-Emotional Problems.

Variable	*B*	95% CI for *B*		*SE B*	*β*	*R* ^2^	Δ*R^2^*
		*LL*	*UL*					
Step 1						.00	.00
Intercept	2.05	1.97	2.14				
Coaching experience	0.00	0.00	0.01	0.00	0.03		
Step 2						.04	.04
Intercept	1.59	1.29	1.89				
Coaching experience	0.00	0.00	0.01	0.00	0.03		
Child gender	0.00	−0.12	0.10	0.06	0.00		
Age group	0.03	−0.01	0.07	0.02	0.07		
Shyness	0.01	0.01	0.02	0.00	0.18		
Step 3						.04	.00
Intercept	1.59	1.29	1.89				
Coaching experience	0.00	0.00	0.01	0.00	0.03		
Child gender	0.00	−0.12	0.10	0.06	0.00		
Age group	0.03	−0.01	0.07	0.02	0.07		
Shyness	0.01	0.01	0.02	0.00	0.18		
Shyness × age group	0.00	−0.06	0.05	0.03	−0.01		
Shyness × child gender	0.01	−0.05	0.07	0.03	0.02		

Results for *beliefs of sports problems* are presented in Table 4. The linear regression indicated that there was no significant association between coaches’ shyness and their belief that shy athletes will experience sports problems in the team context, while holding child gender and age coached constant *(b* *=* .007*, p* = .143)*.* That is, coaches’ shyness did not significantly predict the belief that shy athletes will experience sports-related problems. No other significant main or interaction effects were found.

**Table 4.** Hierarchical Regression Results for Beliefs of Sports Problems.

Variable	*B*	95% CI for *B*		*SE B*	*β*	*R* ^2^	Δ*R^2^*
		*LL*	*UL*					
Step 1						.00	.00
Intercept	2.19	2.05	2.30	0.06			
Coaching experience	0.00	−0.01	0.01	0.00	−.03		
Step 2						.02	.02
Intercept	1.71	1.39	2.21	0.21			
Coaching experience	0.00	−0.01	0.00	0.00	−.05		
Child gender	0.10	−0.01	0.23	0.08	.07		
Age group	0.04	−0.01	0.10	0.03	.07		
Shyness	0.01	0.00	0.01	0.01	.07		
Step 3						.02	.00
Intercept	1.70	1.38	2.20	0.21			
Coaching experience	0.00	−0.01	0.00	0.00	−.05		
Child gender	0.10	−0.08	0.23	0.08	.07		
Age group	0.04	−0.01	0.10	0.03	.07		
Shyness	0.01	−0.01	0.01	0.01	.08		
Shyness × age group	0.00	−0.09	0.06	0.04	−.01		
Shyness × child gender	−0.01	−0.09	0.06	0.04	−.01		

## Discussion

The goal of this study was to examine links between coaches’ shyness and their perceptions of shy and verbally exuberant children in a team sports context. Overall, results indicated that coaches in general tend to worry more about shy children compared to verbally exuberant children. However, findings also suggest that coaches’ shyness is associated with greater worry and anticipation of peer problems for athletes with behavior problems, as well as lower self-efficacy for working with these athletes. Further, results indicated that coaches’ own shyness predicted the belief that shy athletes will experience socio-emotional problems, but not sports-related problems.

### Beliefs About Shy vs. Exuberant Youth Athletes

Our results indicated that, in general, there were minimal differences in coaches’ beliefs about shy versus exuberant youth athletes. Specifically, results indicated a significant difference between vignettes only for worry, in that coaches were more likely to worry about hypothetical shy athletes than hypothetical exuberant athletes. This finding is in line with results from Kirkpatrick et al. (2020), in which undergraduates with coaching experience reported significantly greater worry towards hypothetical shy athletes than exuberant athletes. This suggests that coaches may be more likely to act overprotectively towards shy athletes, which may reinforce or exacerbate athlete shyness.

No significant differences emerged between the vignettes regarding coaches’ feelings of anger. This contrasts with findings from Kirkpatrick et al. (2020), as their results found that coaches reported significantly more anger towards hypothetical exuberant athletes than shy athletes. However, Kirkpatrick et al. (2020) studied a sample of undergraduate students, whereas our sample was comprised of older coaches. It can be speculated that older coaches would be less likely to respond with overt anger to athlete's behaviors, given that older adults display more often use calming strategies to control feelings of anger when compared to younger adults (Phillips et al., 2006).

Additionally, there were no significant differences between the vignettes regarding coaches’ anticipation of negative social outcomes for youth athletes. This also contrasts with findings from Kirkpatrick et al. (2020), as their results found that coaches anticipated negative social outcomes for hypothetical shy athletes more than exuberant athletes. As detailed above, differences in our samples may mean that the current study included older coaches with more experience. These coaches appear to believe that both shyness and exuberance could lead to negative social outcomes for athletes. This somewhat unexpected finding will need to be explored further in future research.

Finally, there were no significant differences between vignettes regarding coaches’ self-efficacy for working with youth athletes with different behavioral tendencies. Although this variable has not been explored in the few previous studies on coaches’ perceptions towards athletes with unique behavioral tendencies, these initial findings suggest that, in general, coaches do not believe that shy athletes are more difficult to work with than exuberant athletes, or vice versa.

### Coaches’ Shyness

The results of the current study found that coaches’ self-reported shyness predicted greater worry towards both shy and verbally exuberant children, the stronger belief that these children will experience peer and socio-emotional difficulties, and lower self-efficacy for working with such children in the team sports context. These findings are in keeping with previous research indicating that shyness among teachers is associated with lower self-efficacy for working with both shy and verbally exuberant students (Deng et al., 2021). Additionally, our findings are in line with broader research suggesting that shyness in adulthood is associated with lower confidence and self-esteem (Bober et al., 2022). However, particularly with regard to the findings for self-efficacy, it should be noted that effects sizes were modest. As such, although shy coaches may have tended to express lower self-efficacy than their more sociable counterparts, it is not necessarily the case that shy coaches have “low” self-efficacy.

Notwithstanding, taken together, these results suggest that shy coaches may be prone to both worrying about the experiences of athletes displaying unique behavioral tendencies—and feeling less confident in being able to effectively coach such athletes. As such, additional psychoeducational training for coaches pertaining to working with a variety of personality and behavior types is warranted. Such training may also be particularly helpful for coaches who tend to be more shy to boost their confidence in their abilities to provide a positive environment for youth athletes and bolster their behavioral management skills. For example, previous research suggests that the negative association between shyness and self-esteem is mediated by issues related to self-presentation (Bober et al., 2022). As such, shy coaches be helped to adopt a more self-promoting behavioral style to their coaching, which could foster their self-efficacy in behaviorally challenging situations.

However, contrary to our hypotheses, no significant interactions were found between coaches’ shyness and the type of vignette. That is, coaches’ shyness was associated with the outcome variables regardless of the type of behavioral tendency of the child in the hypothetical vignette. These results suggest that shyness in adulthood may manifest itself as increased perception to social threat and the tendency to negatively interpret ambiguous social situations (Tang et al., 2016). Longitudinal research has found that individuals who were shy in childhood and continue to experience shyness in adulthood are at greater risk for developing clinical social anxiety, compared to those whose childhood shyness decreased into adulthood (Tang et al., 2017). Further, these same individuals who maintained their shyness into adulthood were found to have a greater attentional bias towards faces displaying negative emotions than those low in shyness in adulthood (Tang et al., 2017). Our findings extend this research by providing evidence that *coaches’* shyness is associated with the anticipation of negative outcomes for youth who display unique behavioral differences in a *team sports* setting. Previous research has suggested that parents who anticipate greater risk for and worry about their shy children are more likely to be overprotective (Coplan et al., 2009) which may lead to greater problems for shy children (Coplan et al., 2008). Considering the current study's findings, it may be the case that shy coaches also act overprotectively towards their youth athletes, which could lead to further behavior difficulties, particularly for shy athletes.

Coaches’ shyness also predicted lower perceived self-efficacy for working with behaviorally unique youth. This finding aligns with the literature on shyness in adulthood. For example, shy individuals tend to experience relatively low general self-efficacy, which is suggested to lead to lower subjective well-being overall (Baker & McNulty, 2010). Further, individuals who are shy often experience lower confidence and self-esteem than their more sociable counterparts (Bober et al., 2022). In particular, shyness is associated with lower confidence in one's social communication abilities (Iranmanesh et al., 2021). As such, shy coaches may be more likely to perceive that they do not have the ability to respond competently to youth athletes who display challenging behaviors.

### Limitations and Future Directions

This study, although novel in its examination of coaches’ shyness, is not without its limitations. To begin, although we attempted to recruit a diverse sample, this was not a random or representative sample of coaches. We should thus be cautious about the generalizability of our findings. Related to this, effect sizes for the present study were also generally quite small, further raising potential issues about the impact of these findings. As well, this study requires participants to imagine coaching a type of child that they may have never encountered before. Given that we did not measure participants’ ability to immerse themselves in an imaginative scenario, they may not have been able to completely accurately report how they would feel and respond towards each hypothetical child athlete. Future research should include this measure when having participants respond to the hypothetical vignettes. As well, although converging evidence from previous studies suggests that teachers (Coplan et al., 2011) and undergraduate students with coaching experience (Kirkpatrick et al., 2020) differentiate between the hypothetical shy and verbally exuberant children as described in these vignettes, further validation of this measure (including a direct manipulation check) remains warranted.

It is also the case that findings regarding the newly developed *Potential Problems for Shy Athletes* scale should be considered as preliminary in nature. This new measure displayed a theoretically consistent factor structure and evidence of internal reliability in the current sample. Coaches’ shyness was positively associated with the belief that shy athletes would display socio-emotional problems, which also provides some initial evidence of construct validity. Notwithstanding, this new assessment requires additional validation in a wider range of samples and contexts.

Future research may also benefit from taking a qualitative or mixed methods approach by asking participants to detail their personal experiences working with shy or verbally exuberant youth athletes. The current study is limited in that we used structured survey questions to investigate participants’ experiences, which may only allow for surface-level responses. Future qualitative studies in an open-ended format would allow researchers to dive deeper into the experiences of shy coaches. Further, conducting observations of coaches’ behaviors during practice while interacting with youth athletes would allow for the collection of more in-depth, accurate data regarding how coaches respond to different behaviors. Given the lack of confidence often experienced by shy individuals, they may underestimate their abilities in questionnaire-based studies. Observational studies would allow researchers to more objectively assess shy coaches’ responses to their athletes in naturalistic settings.

Second, this study only considers shyness as a personality trait that may influence coaches’ perceptions towards youth athletes. However, other traits may be particularly beneficial or detrimental in the context of youth sports. As such, future studies should consider the influence of additional traits on coaches’ perceptions towards youth athletes with certain behavioral tendencies, such as competitiveness and agreeableness, as well as characteristics that share some conceptual overlap with shyness, such as introversion and neuroticism. Similarly, future research would benefit from exploring the influence of coaches’ personalities on their perceptions towards a wider range of child social behaviors, such as aggression or unsociability. Relatedly, the current study does not consider the influence of specific sports contexts on coaches’ perceptions towards different athlete behaviors. As such, future research should explore differences among these different contexts such as elite versus competitive versus recreational sports, and different types of team sports (Blain et al., 2025).

Finally, the current study is limited in that it does not consider the perceptions of shy and verbally exuberant athletes towards their coaches. That is, although our results suggest that shy coaches may be at a disadvantage when coaching youth with unique behavioral tendencies due to worry, low self-efficacy, and the anticipation of negative outcomes, real youth athletes may not necessarily be affected by this. As mentioned above, a personality fit between an athlete and their coach may actually be of benefit to reaching common performance-related goals (Stanford et al., 2022). To better understand the effects of a coaches’ personality on their shy athletes, future studies should consider recruiting entire sports teams.

## Conclusion

Despite the limitations of the current study, our findings add to the existing literature on coaches’ perceptions towards shy athletes by considering the effect of coaches’ own personalities. These findings have potential implications in the domain of education and training for coaches, which may ultimately improve the experience of team sports for shy and exuberant youth athletes.

## Notes

1. Results from additional analyses revealed no significant differences in coaches’ shyness as a function of the gender of the coach (male vs. female) or the gender of the teams coached (boys vs, girls vs. both). As such, these variables were not controlled for in subsequent analyses.

2. Although this response option was included as an option, it was not selected by any participants

## Appendix A

### Child Behavior Vignettes

Instructions: As you read each of the three vignettes below, please imagine that it is a child on your team being described. After each story, there are eight questions describing attitudes or beliefs you may have if you saw a child behaving that way. Maybe you have had players similar to those described in the vignettes, and you can remember how you felt and what you did. If you have never had a player like one described in a scenario, please imagine what it would be like to be in that situation and see the child behaving this way. Please try to answer every question. If you are not sure about an answer, just give us your best guess. Also, we are interested in what you think, so please do not talk about the scenarios and questions with other people until after you have given us your answers.

#### Boy's Version

- Shyness

Just before the start of practice, Adam is hovering near some other children on his team who are talking and laughing together. Adam appears somewhat anxious. He inches closer to the other children, but does not try to join in.

- Verbal Exuberance

While you are talking with the team, Murray blurts out answers and frequently interrupts other children. He cannot contain his excitement and tends to speak too loudly and too often.

#### Girl's Version

- Shyness

Just before the start of practice, Anna is hovering near some other children on her team who are talking and laughing together. Anna appears somewhat anxious. She inches closer to the other children, but does not try to join in.

- Verbal Exuberance

While you are talking with the team, Marie blurts out answers and frequently interrupts other children. She cannot contain her excitement and tends to speak too loudly and too often.

## Appendix B

### Potential Problems for Shy Athletes Scale

**Table:**

How often do shy athletes on your team have problems with the following?					
Never	Sometimes	About half the time	Usually	Always	Don’t know^2^
1	2	3	4	5	6
*Social emotional problems* 1. Making friends 2. Being teased by other team members 3. Having lower self-esteem 4. Trying to participate but not succeeding 5. Being lonely					
*Sports problems* 1. Achieving basic playing skills 2. Performing up to skill level 3. Working as part of a team					
